# Disentangling the effects of a multiple behaviour change intervention for diarrhoea control in Zambia: a theory-based process evaluation

**DOI:** 10.1186/s12992-017-0302-0

**Published:** 2017-10-17

**Authors:** Katie Greenland, Jenala Chipungu, Joyce Chilekwa, Roma Chilengi, Val Curtis

**Affiliations:** 1 Department for Disease Control, Faculty of Infectious and Tropical Diseases, London School of Hygiene and Tropical Medicine, Keppel Street, WC1E 7HT, London, UK; 2 Centre for Infectious Disease Research in Zambia, Plot 5032 Great North Rd, Lusaka, Zambia

**Keywords:** Process evaluation, Theory of change, Behaviour change, Handwashing, Breastfeeding, Oral rehydration salts, Zinc

## Abstract

**Background:**

Diarrhoea is a leading cause of child death in Zambia. As elsewhere, the disease burden could be greatly reduced through caregiver uptake of existing prevention and treatment strategies. We recently reported the results of the *Komboni Housewives* intervention which tested a novel strategy employing motives including *affiliation* and *disgust* to improve caregiver practice of four diarrhoea control behaviours: exclusive breastfeeding; handwashing with soap; and correct preparation and use of oral rehydration salts (ORS) and zinc. The intervention was delivered via community events (women’s forums and road shows), at health clinics (group session) and via radio. A cluster randomised trial revealed that the intervention resulted in a small improvement in exclusive breastfeeding practices, but was only associated with small changes in the other behaviours in areas with greater intervention exposure. This paper reports the findings of the process evaluation that was conducted alongside the trial to investigate how factors associated with intervention delivery and receipt influenced caregiver uptake of the target behaviours.

**Methods:**

Process data were collected from the eight peri-urban and rural intervention areas throughout the six-month implementation period and in all 16 clusters 4–6 weeks afterwards. Intervention implementation (fidelity, reach, dose delivered and recruitment strategies) and receipt (participant engagement and responses, and mediators) were explored through review of intervention activity logs, unannounced observation of intervention events, semi-structured interviews, focus groups with implementers and intervention recipients, and household surveys. Evaluation methods and analyses were guided by the intervention’s theory of change and the evaluation framework of Linnan and Steckler.

**Results:**

Intervention reach was lower than intended: 39% of the surveyed population reported attending one or more face-to-face intervention event, of whom only 11% attended two or more intervention events. The intervention was not equally feasible to deliver in all settings: fewer events took place in remote rural areas, and the intervention did not adequately penetrate communities in several peri-urban sites where the population density was high, the population was slightly higher socio-economic status, recruitment was challenging, and numerous alternative sources of entertainment existed. Adaptations made by the implementers affected the fidelity of implementation of messages for all target behaviours. Incorrect messages were consequently recalled by intervention recipients. Participants were most receptive to the novel disgust and skills-based interactive demonstrations targeting exclusive breastfeeding and ORS preparation respectively. However, initial *disgust* elicitation was not followed by a change in associated psychological mediators, and social norms were not measurably changed.

**Conclusions:**

The lack of measured behaviour change was likely due to issues with both the intervention’s content and its delivery. Achieving high reach and intensity in community interventions delivered in diverse settings is challenging. Achieving high fidelity is also challenging when multiple behaviours are targeted for change. Further work using improved tools is needed to explore the use of subconscious motives in behaviour change interventions. To better uncover how and why interventions achieve their measured effects, process evaluations of complex interventions should develop and employ frameworks for investigation and interpretation that are structured around the intervention’s theory of change and the local context.

**Trial registration:**

The study was registered as part of the larger trial on 5 March 2014 with ClinicalTrials.gov: NCT02081521.

**Electronic supplementary material:**

The online version of this article (10.1186/s12992-017-0302-0) contains supplementary material, which is available to authorized users.

## Background

Promotional strategies designed to improve behaviours related to health conditions differ widely in their theoretical grounding, content, mode of delivery and effectiveness [1–4]. This makes it difficult to know which approaches are most worthy of future investment. For interventions to improve we need scientific advance, both through the development of innovative approaches and also by learning from them through evaluations that do not just measure outcomes but also seek to explain them. [5, 6]. These are so-called ‘Process Evaluations’ [7]. However, there is an acute lack of published studies demonstrating how methods recommended in the literature have been employed in process evaluations [8–10]. This shortage of examples constrains application of these methods to the evaluation of real interventions. Here we present a theory of change based process evaluation of an intervention designed to change multiple behaviours related to childhood diarrhoea in Zambia..

There are many proven low cost household interventions for the control of childhood illnesses, however, low rates of caregiver uptake and lack of compliance mean that their health impact is often limited [11–17]. For example, behaviours such as exclusive breastfeeding and handwashing with soap are known to protect against childhood diarrhoea [18–20], whilst treatment outcomes are improved by the use of oral rehydration salts (ORS) and zinc supplementation [21, 22]. However, despite many years of efforts, caregiver uptake of these practices remains low or inconsistent [11, 16, 20, 23, 24].

The *Komboni Housewives* was an innovative intervention designed to encourage change in diarrhoea control behaviours in mothers of children under-5 years-of-age in Lusaka Province in Zambia. *Komboni* means compound, which describes the informal settlement areas which are typical of urban Zambia. The intervention promoted four behaviours: the exclusive breastfeeding of infants up to 6 months-of-age; handwashing with soap after defecation; the correct preparation and use of oral rehydration salts (ORS); and zinc supplementation during the home management of diarrhoea.

A cluster randomised trial of this complex intervention showed that the proportion of infants aged 0–5 months reported to be exclusively breastfed improved from 39% at baseline to 61% 4–6 weeks post-intervention, a baseline and age-adjusted difference of +11% compared with the control group (*P* = 0.03). Zinc awareness was significantly higher in the intervention group post-intervention (+25%, 95% confidence interval (CI) 11% to 39%). The intervention had no measurable impact on handwashing with soap practices, correct preparation of ORS solution or reported use of ORS and zinc for home treatment of childhood diarrhoea. However, exploratory analysis suggested that all target behaviours improved in the intervention clusters which had the highest levels of exposure, with significant improvements in exclusive breastfeeding (+14%, 95%CI 3% to 25%) and the method of ORS preparation (+16%, 95%CI 2% to 27%) only. The full results from the outcome evaluation are reported in the main trial paper [25].

Here we describe the results of a process evaluation designed to understand the success and failure of this complex intervention targeting multiple behaviours in a challenging context. The aim was to discover if the intervention’s lack of success was related to the underlying theory of change, to problems in delivery, or to problems in uptake. The implications for the future of the *Komboni Housewives* intervention, for process evaluations and for intervention science are also discussed. Below we being with a brief description of the intervention and its theory of change.

### The *Komboni Housewives* intervention

The intervention was designed following the design stages and underlying theory of behaviour of the Behaviour Centred Design approach [26]. The approach was employed as follows: we reviewed knowledge about the behaviours in question, both internationally and locally; we conducted formative research to expand on this knowledge [27]; finally, we used formative research findings and the past experience of the investigators [28–30] to develop a theory of change for the intervention and we engaged a local creative agency *(DDB Iris)* to develop the intervention. Intervention concepts and materials were piloted in focus groups with caregivers of children under-five to test comprehension, relevance and acceptability. The intervention was delivered from March to September 2014 and was evaluated through both an outcomes evaluation (reported elsewhere [25]) and process evaluation (reported here).

The theory of change of the intervention was founded on the insight that people in this populous social context care about their social reputation and seek to avoid becoming the subject of adverse local gossip (the *affiliation* motive [31]). A fictional group of amiable, gossipy local characters known as the *Komboni Housewives* was deployed to suggest that practicing the target behaviours would lead to social approval (the underlying theory of change). The overarching goal was thus to create an environment where mothers would expect that other mothers would notice and approve when they behaved correctly with respect to the four behaviours. Actors playing the *Komboni Housewives* held women’s forums in the homes of caregivers of children under-five, facilitated radio call-in programmes during these forums, and co-led road shows featuring a famous Zambian musician. Daily clinic sessions at the ORT (oral rehydration therapy) corner at health clinics were carried out to target mothers at a hypothesised ‘teachable moment’ [32] (when their child was ill) as a complement to community activities. These sessions were run by volunteer health workers (Neighbourhood Health Committee Volunteers – NHCs) affiliated to the health clinics. Radio programmes were hosted by local DJs who were trained and incentivised to discuss the target behaviours and play campaign radio adverts. These programmes were aired in both intervention and control areas (which means that the impact of the radio element on behaviour could not be captured in the primary outcome evaluation).

The intervention also employed other motives (*disgust* and *nurture)* [31] and provided information to enhance knowledge, and to address barriers and misconceptions associated with practice of the target behaviours. Disgust was used in a ‘Baby Tummy’ demonstration which simulated the contents of the stomach of a mixed-fed baby to encourage exclusive breastfeeding. Interactive demonstrations based on the nurture motive were used to convey the functional benefits of administering correctly-prepared ORS, whilst skill in ORS preparation was boosted through demonstration and behaviour modelling. Information to raise awareness of and demand for zinc was provided during all activities involving ORS. An interactive ‘Shit and Shake’ activity [33] sought to heighten disgust associated with not washing hands with soap after toilet use.

Further details on the intervention content and delivery schedule can be found in Table 1 and on the campaign website: http://kombonihousewives.lshtm.ac.uk.

**Table 1:** Overview of intervention content and delivery schedule

Component	Target Audience	Setting	Implementers	Content	Delivery
Radio adverts & Call-in Show	Population in target areas, particularly caregivers of children under-five	Broadcast on 3 radio stations: Komboni Radio, Radio 1 and Radio 4	*Komboni Housewives* and Radio Master of Ceremonies (MCs)	Airing of three different spot adverts (EBF, HWWS, ORS + Zinc); similar content to that described in the forum & road shows skits. Call-in shows used as a discussion forum and to amplify the activities of the women’s forums (the timing of the shows coincided with the women’s forums). Discussions scripted around the target behaviours to test the callers' understanding of the intervention messages. Jingle about the target behaviours also played.	3 times a week for 6 months, with penetration in both intervention and control areas.
*Komboni Housewives* Women’s Forums	~20 caregivers of children under-five	Forums held in the community at the home of a host (an intervention recipient)	*Komboni Housewives*	All four behaviours targeted using: 1) skits (feature the Komboni Housewives gossiping about mothers they believe are not practicing the correct behaviours, being proven wrong and welcoming the mother into their group); 2) discussion with question and answer sessions; 3) emotionally engaging demonstrations (designed to evoke feelings of disgust at mixed feeding a baby under six months and not handwashing with soap, and nurture in relation to incorrect preparation of ORS); and 4) short films featuring the *Komboni Housewives* (introduced partway through the intervention period)*.* Activities were supported by banners, certificates, stickers, a branded bus and prizes (hats and T-shirts).	One or two forums a day throughout intervention period; rotating between the eight intervention areas.
ORT Corner "*Circle of mothers*" sessions (with monthly prize draw)	Caregivers of children under five (preferentially those with a child presenting with diarrhoea)	At the ORT corner (where ORS solution is traditionally available) or another designated area in the government clinic in each intervention area	Two Neighbourhood Health Committee Volunteers (NHCs) linked to the clinic in each site	Circle of Mothers: content similar to forums designed to be shorter and focussed on exclusive breastfeeding and ORS and zinc.	Every Monday-Friday at clinics in all 8 sites.
				Prize draws: Winner of a hamper selected from all caregivers who attended the clinic session in the previous month. The *Komboni Housewives* conducted a mini forum at select prize draws.	Monthly in each site. Attended by *Komboni Housewives* once per site.
Road shows	All community members	Large public space in each site	MCs and *Komboni Housewives* (featuring a well-known local musician, *Afunika)*	Large road shows, one in each intervention area. Similar content to the forums but energised by the presence of the MCs and the presence of *Afunika* who sang the campaign song and engaged the audience in discussion about the target behaviours. CDs featuring the campaign song as well as hat and T-shirts were given to those giving correct answers in a quiz.	One road show in each site.

*MC* Master of Ceremonies, *EBF* exclusive breastfeeding, *HWWS* handwashing with soap, *ORS* oral rehydration salts, *ORT* Oral rehydration therapy

## Methods

### Evaluation design and framework

**Fig. 1:**
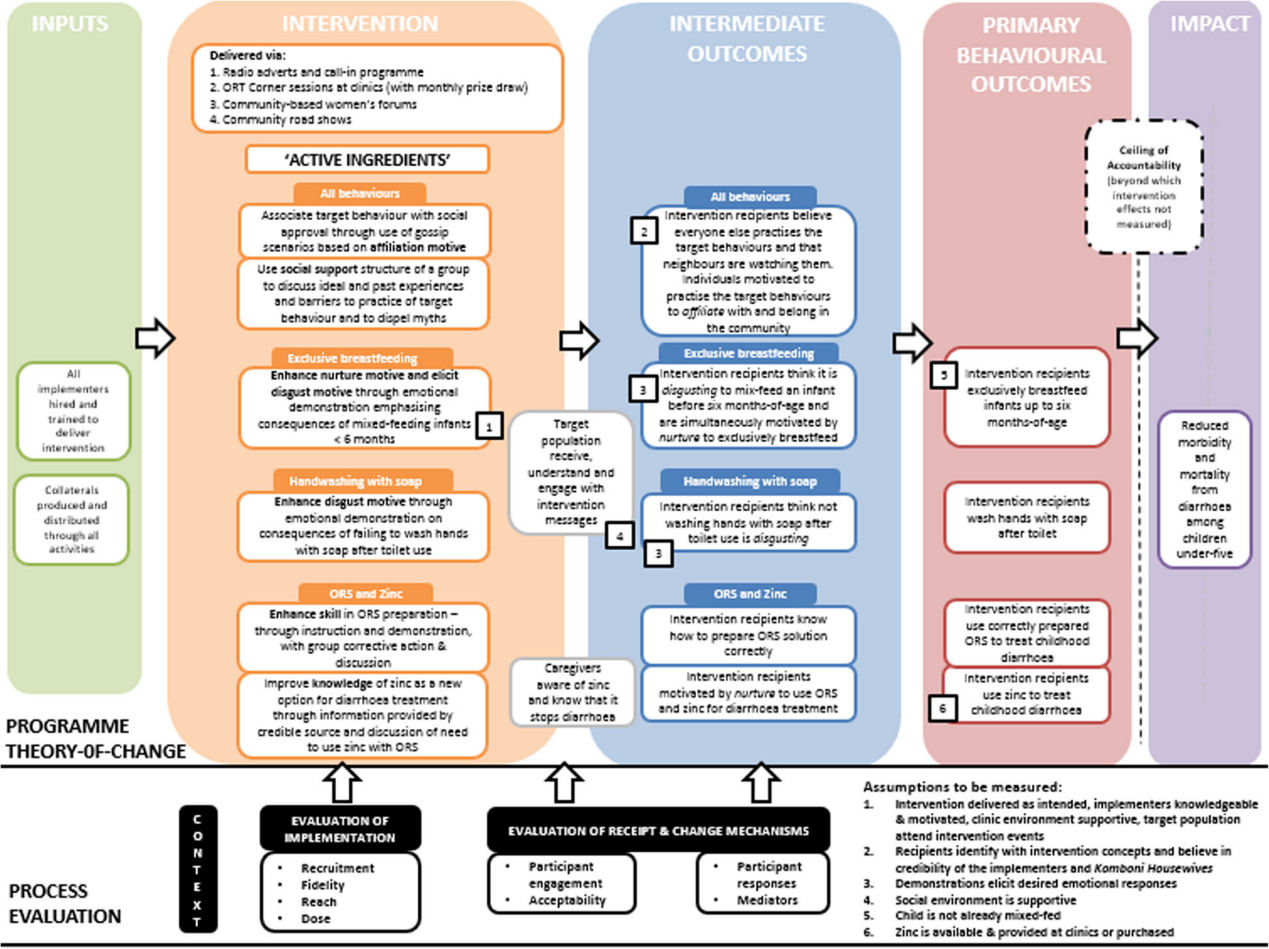
Process evaluation framework

The process evaluation was structured around the theory of change (ToC) for the intervention following the theory-based approach to evaluation [34]. We added eight evaluation ‘domains’ to this ToC, in line with Linnan and Steckler [35] and others [9, 36, 37]. The ToC model depicted in Fig. 1 shows how the intervention’s defined ‘active ingredients’ were hypothesised to act on behavioural determinants (the intermediate outcomes) to bring about change in the four target behaviours, with the ultimate goal of reducing morbidity and mortality from childhood diarrhoea. Assumptions that needed to hold true for change to proceed as predicted are also illustrated. The process evaluation domains are grouped into categories of ‘implementation’, ‘receipt and change mechanisms’ and ‘context’ and the timing of their measurement is shown relative to the ToC: the domains associated with implementation measured aspects of intervention delivery, whilst receipt and mechanisms of change explored the effects of the delivered intervention content (i.e. programme theory). This framework was influenced by recent guidance on process evaluation of complex interventions [9].

The domains were defined as follows:

- *Fidelity -* the content and quality of the implemented intervention compared with what was intended;
- *Dose delivered -* the number of events that were actually conducted;
- *Reach -* the degree to which the target audience participated in the intervention;
- *Recruitment strategies -* the procedures used to attract intervention recipients;
- *Participant engagement & responses -* receipt and understanding of key messages, and interaction with the content;
- *Acceptability -* from the perspective of both the recipients and the implementers;
- *Mediators -* specific behavioural determinants measured along the hypothesised causal pathway;
- *Context* - events and influences in the intervention setting and environment that may have encouraged or impeded intervention delivery, receipt and uptake.

### Data collection

Data were collected from the eight intervention areas throughout the six-month intervention period to assess intervention implementation and participant engagement. Activity logs were completed at all events and a total of 48 observations of delivered events, 29 post-event interviews with pairs of recipients and 4 interviews with implementers took place. Further data were collected from all 16 intervention and control areas at endline, 4–6 weeks following the termination of activities, comprising 820 household surveys (491 in intervention clusters and 330 in control clusters), 17 semi-structured interviews with intervention recipients, 10 focus group discussions with the *Komboni Housewive* implementers (*N* = 1), intervention recipients (*N* = 6), and intervention non-recipients (*N* = 3) and interviews with all eight pairs of NHC implementers and the Nurse-In-Charge at each intervention clinic. Table 2 summarises the data collected during the process evaluation. Each method is described in more detail in the text below. Tool development was guided by examples in the literature [35, 38–41] and the research needs. All tools were piloted prior to their use.

**Table 2:** Overview of process evaluation methods

Research Method or Data Source	Data Type	Respondents	Core Information Sought	Purpose of Information	Timing
Activity logs	Quantitative	NHC & *Komboni Housewife* implementers	Number of individuals from target population attending Forums and ORT Corner Sessions. Number of men, women and children attending Road Shows. Activities conducted, availability of supplies and challenges faced	Fidelity, dose delivered, reach	Throughout intervention
		CIDRZ staff	Content and quality of delivery of Radio shows	Fidelity	Throughout intervention
Spot check field observations	Quantitative	CIDRZ staff	Content and quality of delivery and participant engagement, according to an observation checklist. Contextual information on delivery & receipt in clusters, including features of each site.	Fidelity, participant engagement, context	Throughout intervention
Semi-structured interviews	Qualitative	NHC & *Komboni Housewife* implementers	Successes and challenges of intervention delivery from perspective of the implementers Recruitment strategies and challenges in each cluster (to enable comparison with the levels of reach achieved in that cluster) Acceptability of intervention messages and activities	Fidelity, recruitment, context, acceptability, participant engagement & responses	Midway through intervention & 4–6 weeks post intervention
		Creative Agency (DDB) and Activation Agency (EXP)	Reasons for any deviations from planned activities	Fidelity	Midway through intervention
		Nurse-in-Charge at intervention clinics	Information on the (clinic) environment and work load of staff Health Centre perspective on ORT Corner Sessions	Context, acceptability	4–6 weeks post intervention
		Intervention recipients (pairs)	Comprehension of messages and emotional responses to the intervention. Acceptability of intervention messages and activities	Participant responses, acceptability	Throughout intervention
		Intervention recipients	Retention of key messages and reflections on the intervention	Participant responses	4–6 weeks post intervention
Focus group discussions	Qualitative	*Komboni Housewife* implementers	Successes and challenges of intervention delivery from perspective of the implementers Recruitment strategies and challenges in each cluster (to enable comparison with the levels of reach achieved in that cluster) Data on delivery & receipt in clusters, including features of each site Acceptability of intervention messages and activities	Fidelity, recruitment, context, acceptability, participant engagement & responses	4–6 weeks post intervention
		Intervention recipients	Retention of key messages and reflections on the intervention. Reactions to gossip in relation to the target behaviours	Participant responses, mediators	4–6 weeks post intervention
		Unexposed control arm participants	Reactions to gossip in relation to the target behaviours	Mediators	4–6 weeks post intervention
Household survey	Quantitative	Sample of individuals in intervention arm and control arm	Proportion of sample reporting attendance of each intervention component/listening to the radio show at least once in each intervention and control cluster. Recall and recognition of intervention concept, messages. Quantitative capture of indicators relating to hypothesised behavioural determinants	Reach, participant responses, mediators	At baseline (mediators) & 4–6 weeks post intervention
Document review	Quantitative	Intervention Schedule Spreadsheet	Information on deviations from planned activities	Fidelity	4–6 weeks post intervention

NHCs (Neighbourhood Health Committee volunteers) implemented the intervention at the clinics (ORT Corner Sessions), the hired actors (the Komboni Housewives) implemented the forums and road shows and were managed by EXP (an Activation Agency)

#### Activity logs

Implementers kept records of attendance, the availability of supplies and any challenges or unexpected incidents that affected the delivery of all intervention activities. Attendance was measured by head count shortly after the start of each event. A media monitoring log was used to capture whether the radio call-in programme had taken place and to record any deviations from the intended content.

#### Field observations

A trained evaluator periodically carried out unannounced field visits throughout the intervention period to observe implementation of ORT corner sessions (*N* = 30), forums (*N* = 10) and each road show (*N* = 8). A structured reporting form was used to record details about the setting, fidelity according to criteria related to adherence to the protocol, the competence of delivery and participants’ reactions to the event [42, 43]. Technical problems, attendance and perceived participant engagement were also captured. Following observation, feedback was provided to implementers to improve intervention fidelity.

#### Interviews

A random sample of 29 intervention recipients were interviewed in friendship pairs following field observations at face-to-face events. Recipients were questioned on their understanding and acceptance of the main messages and activities.

A further 17 intervention recipients were also interviewed following the end of the intervention. These individuals were selected from household survey participants (described below) who reported having attended an ORT corner session, forum or road show. These semi-structured interviews followed a topic guide to explore recall of the intervention content, retention of key messages, and, from the recipients’ perspectives, any lasting impacts of the intervention on beliefs or actions. These interviews also explored whether the central concept of the intervention had been effectively communicated, i.e. whether participants felt that practice of the target behaviours led to social approval, and that individuals who do not practice the target behaviours are the subject of gossip.

Semi-structured interviews conducted during and following intervention delivery with the implementers (eight NHC pairs and a *Komboni Housewives* implementer), the creative agency (*DDB Iris*), the implementing agency (*Exp*) and the coordinating body (CIDRZ) explored intervention management, recruitment strategies and challenges, and fidelity of delivery. Acceptability was explored through questions about the activities the implementers enjoyed delivering or found repetitive or boring, as well as their opinion on their working conditions and job satisfaction. Implementers were also asked how attentive the target population was during the sessions, and whether they thought that the intervention was acceptable from the recipients’ perspective. The head nurse at each intervention clinic was also interviewed to understand how the ORT corner sessions affected other clinic activities.

#### Household survey

Household surveys were conducted at baseline and 4–6 weeks after the intervention in all intervention and control areas. Eligible caregivers (with a child under six-months or a child under-five with diarrhoea) were randomly selected within each cluster with the primary purpose of measuring behavioural outcomes [25]. Household surveys were also used to collect data on attendance at, and recall of, intervention activities and on basic demographic variables. Endline survey participants were shown a campaign sticker, logo and photo of the *Komboni Housewives* and were asked whether or not they had heard of the intervention and what they knew about the topics that were discussed. Questions on the intervention content were unprompted, and thus measured message retention through topic recall rather than recognition. Several Likert-type questions with five response categories (strongly agree, agree, disagree, strongly disagree, don’t know) were included to measure behavioural determinants, or ‘mediators’ specified in the ToC that the intervention aimed to influence, for example, *“I think my neighbours would gossip about me if I did not know how to prepare ORS correctly*”.

#### Focus group discussions

Nine focus group discussions were held with intervention recipients and with unexposed individuals in the control arm. Each focus group included six to eight female caregivers of a child under-five. These individuals were identified through the household surveys and were included on a first-come, first-serve basis. All focus groups explored social norms and opinions on the importance of gossip and social approval and their role in determining perceptions and practice of the target behaviours. The focus groups held with intervention recipients also explored perceptions of the *Komboni Housewives*.

A focus group discussion involving the *Komboni Housewives* actors was carried out to explore deviations from the protocol, recruitment strategies and acceptability.

### Data handling and analysis

#### Quantitative data

Paper records of field observation forms and implementer logs were entered into MS Excel for analysis. Data on intervention exposure and behavioural determinants obtained through the household surveys were cleaned and analysed in *Stata* 14 (StataCorp 2015, College Station, TX, USA). The proportion of survey respondents agreeing or strongly agreeing with Likert-type response questions concerning potential mediators was analysed at cluster-level and compared on an intention-to-treat population involving intervention and control arm participants following the two-step approach recommended by Hayes & Moulton [44]. A single database was created from all the activity logs. The number of events held (dose delivered) and participants in attendance at these events were computed over time and by cluster and intervention component. Reach was computed as the proportion of endline survey respondents reporting attendance at one or more face-to-face event. Reach was computed by wealth tertile (poorest, middle and least poor), which was assessed through principal component analysis of 13 household assets (ownership of home, television, mobile telephone, land for farming, non-domestic animals, car, fridge, freezer, bicycle, radio, water tap inside the home, electricity, flush latrine) and the material of the structure of the floor, roof and exterior walls [45]. A matrix was created to organise fidelity data on adherence and delivery competence by target behaviour, intervention component and cluster.

#### Qualitative data

All interviews and discussions followed a guide and were voice recorded and transcribed *verbatim,* then analysed thematically following the six-step method of Braun and Clarke [46]. Complete transcripts were first read several times and initial impressions about the data were noted. Transcripts were then coded according to pre-specified themes related to the eight evaluation domains. Identified sub-themes were specified for the four target behaviours and the face-to-face intervention components. Following indexing, a series of matrices was created in MS Excel to review data by theme and sub-theme. A Word Cloud was created to represent participants’ reactions to the ‘Baby Tummy’ demonstration using the ‘WordItOut’ online word cloud generator. A model of the proposed causal mechanism was then developed to suggest how intervention effects for each target behaviour were influenced by the fidelity of implementation, participant engagement and participant responses.

## Results

Evaluation findings pertaining to intervention implementation (dose delivered, reach, recruitment and fidelity) and receipt (acceptability, engagement and responses) are presented in turn. Examination of contextual factors affecting intervention implementation and receipt is limited to the physical and demographic characteristics of the clusters and is included in each section as relevant.

### Intervention implementation

Overall, 253 of 489 (52%) surveyed individuals in the intervention arm had heard of the Komboni Housewives campaign, compared with 48 of 330 (15%) control arm participants. The radio show was reportedly heard at least once by 35% of individuals in the intervention arm and 20% in the control arm, and 39% of 493 intervention arm participants reported attending at least one face-to-face intervention component. Fifty-five (28%) of intervention recipients attended more than one event, but only eight individuals attended all three face-to-face events. Intervention delivery differed by intervention component and across clusters.

#### Delivery of intervention components

A total of 1386 ORT corner sessions were held at clinics with 9444 caregivers of a child under-five living within the clinic catchment area. These events were attended by an estimated 12% of the target population. The dose delivered and consequently the total number of intervention recipients was lower than the intended two sessions per site per day (14,000 recipients) because lower footfall in rural clinics meant that on average only one session took place per day. As the ORT corner session was manned by the NHCs on a daily basis, the criteria for recruitment at ORT corner sessions were broadened partway through the intervention period to better utilise resources and increase the number of participants at each event. The recruitment strategy was altered to include all caregivers of children under-five presenting at the clinic, rather than just those with a child with diarrhoea. The alteration to the recruitment strategy increased the dose delivered three-fold in the second half of the intervention period. The quality of the sessions was also inadvertently improved (Quote 1).

*Quote 1:* “*When it was just the diarrhoea cases the programme wasn’t flowing well, but when we included non-diarrhoea cases then it was perfect. The participation was poor when they were few; they would be shy or just concentrating on the child. When there were a lot, one mother would ask a question, the other would rephrase and others would attempt to answer. There was a change in all the corners, you find there was a deeper understanding and the information was spreading vastly though the community. The discussions had improved. When you have a lot of people they even strive to be the one to demonstrate [how to make ORS].” (NHC Implementer).*

*Komboni Housewives* Women’s Forums took place in a host’s home within the community in the intervention areas, thus although the *Komboni Housewives* team of implementers remained the same, the setting of and audience at each forum varied from day to day and from site to site. In total, 158 forums were delivered and attended by 2723 women across the eight sites, 96% of whom were from the target population. It is estimated that 18% of the target population attended a forum. Once again, these totals fell short of the planned 194 forums with 4000 participants, largely because it took longer to recruit participants in the most remote areas, where only one forum could be held per day.

The road shows were initially intended to coincide with and complement other aspects of the intervention and to raise awareness of the activities taking place in the community. However, delays in the creation of the films meant that the first road show was not held until 4 months into the six-month intervention period. Nevertheless, all eight road shows were conducted and were attended by approximately 13,600 men, women and children (1200 to 2200 attendees per road show). One in three community members in intervention areas were estimated to have attended a road show, including 18% of target women**.**

#### Delivery to clusters

Cluster-level attendance at one or more face-to-face event ranged from 14% to 66%. The scattered arrangement of villages and the agricultural workload made it logistically more challenging to recruit mothers to attend forums and road shows in rural areas. However, although more events were held in densely-populated peri-urban clusters than in harder-to-access rural clusters, the total target population in rural areas was smaller. Consequently, the overall reach in rural areas (+60% in three of four rural clusters) was considerably higher than in peri-urban slums (14–35%).

**Fig. 2:**
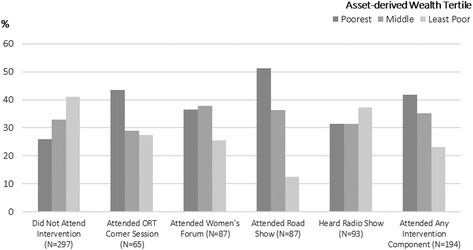
Relationship between socio-economic status and reported intervention attendance

Recruitment was generally easier in densely-populated peri-urban areas. However, implementers noted that it was harder to recruit women to attend forums in two peri-urban clusters with slightly higher socio-economic status and more walled residences. Correspondingly, the poorest individuals in each cluster attended more face-to-face intervention components than their more affluent neighbours (Fig. 2). It appears that the radio programme and road shows, once initiated, helped to increase the legitimacy of the intervention in the eyes of the target population in these less receptive communities (Quote 2).

*Quote 2: “It was such a challenge to do the whole programme and finish it on time in [two clusters]. Basically it was the location; they have no time to waste…. even just bringing the women together was troublesome. That programme on the radio really started changing things. We [also] saw a change after the road shows took place, they really boosted everything. People would start to see us and say: ‘those are Komboni Housewives, when are you people coming to our place?’ I think it helped us a lot because people recognised us.” (Komboni Housewives Implementer)*

The physical layout of a clinic also affected recruitment. Some ORT corner sessions could be held at the site of the existing ORT corner, thus promoting this under-utilised clinic resource. Other clinics were forced to hold the sessions in a separate building that was often harder for participants to locate. The former sessions were often plagued by noise and other distraction that affected session delivery (Quote 3).

*Quote 3: “Since it was an open place people used to move around as you teach so that can make your session bad.” (NHC Implementer)*

Full details of the dose delivered and reach achieved by each intervention component in each cluster can be found in Additional file 1: Table S1.

#### Fidelity of delivery

Implementers appeared to enjoy their work and the status that it afforded, reporting that *“people looked at us like experts”*. In keeping with this, field observations revealed that all implementers liked to educate intervention recipients on topics about which they were knowledgeable. This resulted in the inclusion of additional messages and content on nutrition and on the importance of cleanliness and handwashing before breastfeeding and preparing ORS at ORT corner sessions, forums and road shows (Quote 4).

*Quote 4: “We used to refer them to the clinician if we found that the child is falling under the category of underweight, we wanted the mother or the guardian to have knowledge on how she is going to improve the nutrition status of the child.” (NHC Implementer)*

All implementers also adapted the discussions and skits to try to increase the relevance of the messages for the target audience. The main area where the content was modified was in relation to the skits and discussions around exclusive breastfeeding (Quote 5).

*Quote 5: “You tell the mother to exclusively breastfeed and then she goes ‘what about HIV’ and things like that, and others say ‘what if the milk isn’t coming out, what do you give that child?’ They really wanted us to tell them what to feed that child and so we thought we couldn’t shut them up so we decided to go off [topic] just to keep them, because you know it is not easy to get a woman’s mind to concentrate on what you are saying.” (Komboni Housewives Implementer)*

### Intervention receipt

#### Acceptability, engagement and responses

Intervention recipients most frequently cited being equipped to [teach other women how to] prepare ORS as their favourite aspect of the intervention. Participants interviewed immediately post-intervention had generally understood the main messages, but the target behaviours were not equally recalled: recall of ORS preparation and breastfeeding messages was two to three times higher than recall of zinc and handwashing with soap (although the latter behaviour was not covered at all in the shorter ORT corner sessions) When asked to describe the main message of the event they had just attended, the second most frequently mentioned topic (after diarrhoea avoidance / child health) was ‘cleanliness’. This mirrored the observed emphasis placed on handwashing and cleaning utensils during ORS preparation and the descriptions of the events given by intervention recipients: *“we were learning how to make ORS, washing our hands before doing that and measuring correctly*.”

Message recall assessed during the endline household survey followed a similar pattern to the post-intervention interviews: Handwashing and zinc were mentioned half as many times as breastfeeding, infant feeding, ORS or diarrhoea. The quantitative survey data showed that awareness of zinc as a diarrhoea treatment increased from 25% to 61% as a result of the intervention (adjusted increase of +25% when compared with the control arm, *P* = 0.002). However, interviewed intervention recipients could rarely articulate precisely what zinc was, or how and why it should be use.

**Fig. 3:**
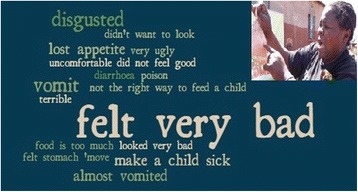
Word cloud illustrating reactions to the ‘Baby Tummy’ demonstration to promote exclusive breastfeeding. *Greater prominence is given to words and phrases that were used more frequently*

Intervention recipients confirmed that they regarded the NHCs and *Komboni Housewives* as knowledgeable and credible information providers. They responded as intended to the ‘Baby Tummy’ demonstration to promote exclusive breastfeeding: the demonstration evoked strong negative disgust-based reactions, with participants most commonly reporting that they ‘felt very bad’, were ‘disgusted’ or thought they would ‘vomit’ (Fig. 3). The Baby Tummy demonstration was well-remembered at endline, but interviews revealed that women more often spoke of the need to stop feeding a child snacks and *“bad”* foods, rather than recalling the messages about exclusive breastfeeding (Quote 6).

*Quote 6:* *“I didn’t know how to prepare porridge in the morning. But after the meeting, I started preparing the porridge.” (InterventionRecipient)*

Consequently, even though disgust was initially elicited following the Baby Tummy activity, individuals in the intervention arm at endline were not significantly more likely to agree with the statement *‘It is disgusting for me to give my baby food or drink before six months’* than control arm participants (49% vs. 43%, *P* = 0.49).

Similarly, the disgust-based handwashing demonstration was not recalled well at endline and intervention arm survey participants found people who do not wash hands after defecation no more disgusting than control arm participants (28% of respondents in both arms agreed that it is disgusting to shake hands with someone who did not wash their hands after using the toilet).

Participant engagement with the intervention in general was affected by the delivery strategy. According to the NHCs, it was sometimes challenging to engage mothers of ill children at the clinic sessions before the recruitment strategy changed (Quote 7). Similar challenges occurred at the other face-to-face events: although mothers were invited in advance to the forums and road shows, it was still difficult to sustain interest throughout these longer events, particularly in areas where the level of formal sector employment was higher. Implementers also felt that the events took place at times when women were busy, which affected their motivation and ability to attend the sessions.

*Quote 7:* “S*ometimes it was difficult, especially if a mother came with a baby that had diarrhoea. We would give them ORS at the corner, but even then you would see that the mother would concentrate on the child rather than listen to what we were discussing.” (NHC Implementer)*

Nevertheless, in the opinion of the implementers, there was demand for the programme from both the participants (Quote 8) and the clinics (Quote 9), although several clinic nurses were concerned that raising awareness of and demand for zinc when the supply is currently limited in the public sector had the potential to cause tension.

*Quote 8: “Other people that stay in far off areas have requested that the programme is extended to reach them so that they also learn how to prepare ORS.” (Komboni Housewives Implementer).*

*Quote 9: “To be frank, we had a bit of gap [in the services we could provide prior to the intervention]: we had a shortage of staff and so we couldn’t really explain to the mothers fully how to give ORS to their children because a nurse had to do it. When the NHCs came they were specifically doing that job unlike a nurse who also had patients waiting for her.” (Head Nurse at an Intervention Clinic).*

Over 90% of survey participants in both study arms agreed that neighbours would gossip about them if they did not take care of their children and a similar proportion agreed that it matters to them if their neighbours gossip about them. Over 70% of survey participants agreed that failing to practise the target behaviours would genuinely incite gossip, but there were no significant differences between the responses given by individuals in the intervention and control arm. When survey participants were asked whether they believed that *their* neighbours would gossip *about them* if they did not practise the target behaviours they were less convinced: 56% of control arm participants and 49% of intervention arm participants agreed that mix-feeding their baby at four or 5 months-of-age would incite gossip (*P* = 0.46); 17% vs. 24% agreed that not knowing how to prepare ORS correctly would lead to gossip (*P* = 0.26); 49% vs. 48% agreed that not washing hands with soap after using the toilet would cause gossip (*P* = 0.88); and 56% vs. 43% agreed that neighbours would gossip if they did not give their child zinc when they had diarrhoea (*P* = 0.45).

However, the specific use of gossip in the intervention (failure to practise the target behaviours) was not well-recalled. Quote 10 is illustrative of the vague answers given by intervention recipients asked to explain the behaviours that the intervention highlighted as promoting gossip.

*Quote 10: “There was a lot of different ways [they talked about gossip], like a woman shouldn’t leave home early in the morning to go and gossip with friends. She just leaves her house work and goes to gossip. A woman needs to work hard at home and cooks food for her children.” (Intervention Recipient).*

Only 30% of respondents in both study arms felt that giving ORS to a child with diarrhoea was common practice in their neighbourhood. However, over two-thirds of participants in both study arms believed that practise of the other target behaviours was already the social norm.

## Discussion

The *Komboni Housewives* campaign was a complex intervention [47], comprising multiple interacting components, targeting four disparate behaviours and applied in diverse peri-urban and rural contexts. This innovative intervention was found acceptable by implementers and the target population alike, and clearly engaged target audiences through its novelty, energy and the appealing *Komboni Housewives* characters that were portrayed. However, the intervention achieved mixed results that are hard to interpret from the cluster-randomised trial results alone [25]. Intervention reach was poor and variable, and behaviour change was limited, except for one reported behaviour (exclusive breastfeeding). Where better reach was achieved, there was an indication that levels of behaviour change were greater, but changes were only significant for breastfeeding and ORS preparation outcomes. The process evaluation collected data on eight domains related to intervention delivery and receipt with the primary aim of explaining why the intervention achieved generally poor and variable levels of behaviour change. The findings are discussed in relation to three key areas: 1) the feasibility of delivering the full intervention to the target population through the chosen delivery channels; 2) the nature and number of behaviours targeted for change; and 3) the motive-centred Theory of Change. The discussion is summarised in a diagram illustrating the proposed mechanisms of change at play in the *Komboni Housewives* intervention.

### Feasibility of intervention delivery

Overall, the programme delivered a lower intervention dose than was intended and this affected its reach and intensity, with only 11% of the surveyed target population reporting attendance at two or more intervention events. As differential reach across sub-groups can bias intervention effects and potentially widen health inequalities [48], it is encouraging that poorer individuals in each community were more frequently exposed to the intervention (and appeared to be more accepting of the intervention content). Radio achieved the best reach (35%), followed by the road shows and forums (both 18%). However, radio covered both intervention and control arms, so its effects could not be assessed. If the radio intervention was effective, this may have diluted the measured levels of behaviour change. The reach of ORT corner sessions was lower (12%), but women throughout the clinic catchment area benefited from this intervention. As each intervention component was delivered in different settings and the intervention duration was short, it is not surprising that relatively few individuals were exposed to the full intervention package. Whilst realistic expectations should be held about the potential levels of reach achievable in interventions delivered in the community [49], sufficient energy also needs to be devoted to the development and tailoring of the delivery channels and recruitment strategies to the intervention setting; the intervention was labour-intensive to delivery in rural areas and did not adequately penetrate communities in several peri-urban sites where the population density was high, the population was slightly more educated and numerous alternative sources of entertainment existed.

### Targeting multiple behaviours

In addition to targeting multiple behaviours, each behaviour consisted of a number of behaviour change tasks. For example, three distinct messages were actually communicated within the messaging on ORS preparation: i) use ORS as soon as a child gets diarrhoea; ii) prepare this ORS solution correctly; and iii) give ORS for the duration of the diarrhoeal episode. Interventions targeting multiple behaviours need to convey clear messages in a consistent way across intervention components. Across the whole intervention, adaptations made by the implementers resulted in the intervention messages losing focus and some of their simplicity, which in turn is likely to have affected message potency [50]. For instance, intervention recipients correctly recalled that they should wash hands and keep utensils clean while preparing ORS, but this added complexity to the existing messages communicated about ORS. It appears that the importance of communicating the specific intervention messages for each behaviour was complex and was not conveyed strongly enough to implementers.

Little is known about how the number, type and sequence of behaviours addressed by multiple behaviour interventions might influence behavioural outcomes and health impact. Proponents of multiple behaviour change interventions argue that reducing several risk factors simultaneously can be more effective in controlling public health problems with multiple causes or multiple transmission routes [51–54]. However, other than their role in diarrhoea control, the four behaviours targeted by the intervention were quite different, taking place at different times and in different places. The process evaluation suggests that the intervention content for the four behaviours was not equal, nor did each behaviour receive equal attention from implementers or the target population. Beyond the fact that handwashing was not included in the intervention delivered at the ORT corner sessions, the handwashing intervention appeared to be particularly ‘light touch’. It is possible that handwashing, which is more commonly the subject of interventions than the other behaviours, received less attention because these messages were delivered alongside other, more innovative, content targeting the other behaviours; the ‘Shit and Shake' brick exercise was never mentioned as an aspect that participants had enjoyed or something that they remembered, suggesting that the activity was either less compelling, or not always implemented. If we also consider that the fidelity of implementation of handwashing messages was low, there are several plausible explanations for the lack of behaviour change in this area.

It is not possible to determine whether the intervention would have been more effective if it had targeted single behaviours, or had targeted each behaviour in sequence, as opposed to simultaneously. However, if we had specified fewer, simpler behaviour change tasks, it is likely that this would have made it easier to communicate, retain and act upon the intervention messages.

### Basing the intervention on *affiliation* and other motives

As well as implementation failure due to low intervention reach and intensity of implementation, and issues with the fidelity of the intervention content concerning the target behaviours, there is indication that programme theory failure was also a problem in this intervention.

Whilst we found that the central concept of the intervention – *affiliation* – was memorable, it did not measurably change norms. This could be due to the low reach of the intervention, a failure to measure norms, or a failure of the central campaign strategy, which was to imply that the target behaviours are normative, likely to be noticed and socially rewarded. It is possible, for example, that in urban areas with low social cohesion [55], individuals do not experience a sense of community and hence are less susceptible to norms-based interventions [56]. Although social cohesion is suspected to have been greater in rural areas, the physical distance between individuals living in villages with scattered housing may have meant that the notion that an individual’s behaviour would be seen by others was implausible. Norms-based interventions have the potential to be powerful [57, 58], but designing interventions to change norms remains a challenge [59]. Injunctive norms – what is commonly approved of and *ought* to be done – may only influence behaviour if they are *salient* for the individual at the time the behaviour takes place [60]; better ways to trigger intervention recall in the settings where behaviour is enacted are thus needed.

There is some indication that the interactive and novel components of the intervention were well-received and more readily recalled by the target audience. It is also possible that the main message was harder to get across during these exciting activities. For example, instead of hearing that they should avoid *all* foods and snacks before an infant turns six-months, intervention recipients seemed to take away the message that they should give their young infants nutritious foods such as porridge instead of snacks (the latter were added to the Baby’s tummy during the demonstration). Nevertheless, *disgust* at mixed-feeding was elicited in response to exposure to the ‘Baby Tummy’ demonstration, and the intervention improved reported practice of exclusive breastfeeding. However, as the intervention group did not report finding it any more disgusting than the control group to mix-feed a baby, the mechanism of change remains unclear.

**Fig. 4:**
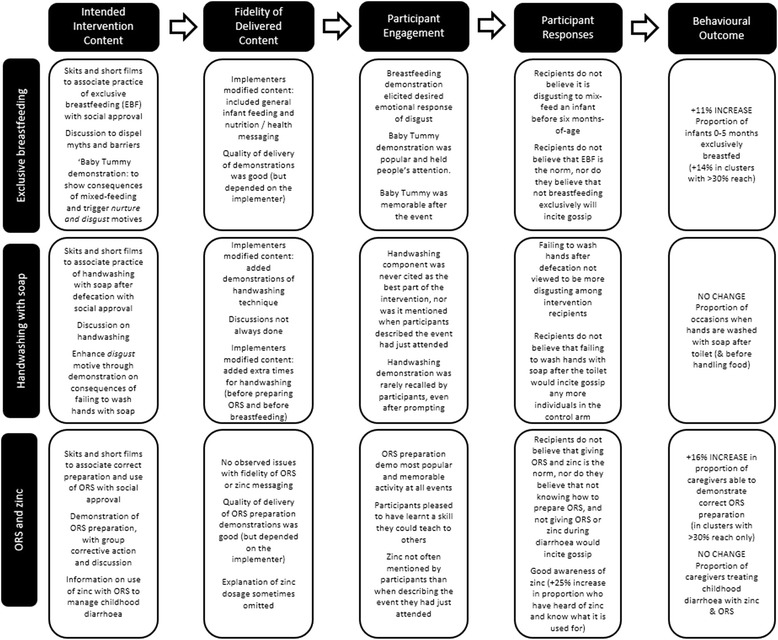
Proposed mechanisms by which the intervention and its implementation influenced behavioural outcomes

The intervention succeeded in enhancing skill in ORS preparation. An unanticipated consequence of this activity was that increased knowledge of a practical, childcare-related skill impacted positively on the intervention’s acceptability to the target population (see Fig. 4). As this intervention component was not technically difficult to deliver, it could be easily adapted and used in other settings. Zinc use, however, was constrained by the limited supply. It is not possible to determine whether increased *awareness* of zinc would have translated to increased *use* of zinc if supply had been widely available at the time of measurement of intervention outcomes. Nor is it possible to know whether use of zinc would have also driven the uptake of ORS – which did not improve, despite improvements in the preparation of ORS solution [25] – as has been demonstrated elsewhere [61].

The handwashing component of the intervention also included a demonstration to elicit *disgust* (the ‘Shit and Shake’ brick activity). However, neither behavioural mediators, nor behaviour itself, changed significantly following exposure to this activity. Formative research findings (unpublished) indicated that the target population were tired of handwashing campaigns. The low implementation fidelity and limited recall of intervention messages by intervention recipients suggest that handwashing messages were indeed unattractive to both implementers and recipients. The formative research also revealed that water, soap and handwashing infrastructure were rarely found together in a convenient place for handwashing, so handwashing behaviour change may also have been limited by the lack of facilities [13, 62–65]. It is also possible that handwashing competed with other more pressing needs in the lives of the target population [66]. However, due to the low implementation fidelity, it is not possible to determine conclusively why the intervention failed to change handwashing behaviour.

Figure 4 depicts the above-described proposed mechanisms of change in the *Komboni Housewives* intervention. The diagram illustrates how intervention outcomes for each behaviour are thought to have been influenced by the way the implemented intervention was received and taken up by the target population.

### Implications for the future of the *Komboni Housewives* intervention

Even though the *Komboni Housewives* intervention did not significantly improve all of the target behaviours, some aspects of the intervention are worth exploring further in a refined intervention. When the target population was reached, the intervention was associated with small improvements in two behaviours: exclusive breastfeeding and ORS preparation. This implies that altering the delivery strategy, so that it achieves greater penetration in all communities, could have the potential to improve the effectiveness of the intervention with respect to these two behaviours. The lack of handwashing behaviour change suggests that the intervention needs considerable revision.

It would be preferable to deliver and evaluate a revised intervention on a smaller scale in *either* rural or peri-urban settings, so that high coverage can be attained and the impact of the intervention on mediators can be better assessed [67]. There is limited consensus on how best to achieve high coverage of public health interventions in low-income countries [68]. The main body of the intervention comprised the community forums delivered by the *Komboni Housewife* actors and the clinic-based ORT corner sessions facilitated by the NHCs. Although not demonstrated by the findings of this intervention, combining facility-based delivery platforms with community-based events has the potential to achieve high intervention coverage [69, 70]. To realise this potential, more implementers would need to be trained to deliver the community-based intervention activities in densely-populated, peri-urban settings. As the network of NHCs reaches a whole community and NHCs are already well-known and respected in the community, use of existing systems for community outreach (such as these NHCs) may result in higher intervention coverage [71].

Further work is required to compare strategies that target single behaviours with those targeting multiple behaviours sequentially or simultaneously, as well as to optimise delivery strategies for community interventions so that they are feasible to implement in both peri-urban and rural settings. It would be helpful to have good measures of ‘implementation strength’ in process evaluations so the intensity of delivery required to achieve health gains can be assessed [72]. Programmers may also need to be more realistic about the costs of achieving high levels of reach for such interpersonal interventions and design studies that are large enough to answer questions about the relative cost-effectiveness of different delivery channels.

### Implications for the future of theory-based process evaluations

The theory of change model for the intervention was useful for thinking through the qualitative and quantitative data that needed to be collected to measure process indicators related to implementation theory (dose delivered, reach, recruitment and fidelity). The framework also helped to ensure that data were collected on key links in the hypothesised pathway to change to assess the programme theory (i.e. participant responses and mediators). However, whilst this research has highlighted the importance of delivering an intervention well, a well-delivered intervention can only change behaviour if the programme theory is sound. Although change mechanisms in the *Komboni Housewives* intervention were explored using mixed methods, the low levels of behaviour change and intervention reach, as well as the difficulties associated with the measurement of some psychological mediators, limited the extent to which the mechanism of change could be elucidated. Despite conducting a comprehensive process evaluation, it was thus not possible to conclude with certainty whether the underlying theory was flawed as well as the delivery strategy.

As it is not possible to predict all possible interactions, tipping points, or potential pathways to change in a complex intervention, we may never be able to fully capture how change has been brought about by an intervention. Nevertheless, a ToC-based process evaluation that seeks to measure key aspects of intervention implementation and receipt, to tease out how the intervention interacts with and is influenced by context, and assesses change mechanisms, brings us closer towards opening up the infamous intervention ‘black box’.

### Limitations and lessons

It is possible that prior knowledge of the outcome of the trial affected our interpretation of the process evaluation data [7, 9]. However, it was important for programmers to have early results concerning outcomes and knowing the results allowed us to direct the analysis of process data towards the outcomes of interest.

Fidelity was assessed primarily through field observation. The evaluator could easily use their checklist to assess – albeit subjectively - the quality of intervention delivery, the absence of any intervention materials, or the unconscious omission of any intervention content. However, it was not possible to assess how the presence of the evaluator affected observed fidelity. The female assessor tried to mitigate the potential influence of her presence by not announcing her arrival and by sitting quietly at the back of the group. She reported that the implementers were not always aware of her presence until she approached them at the end of the session. This suggests that reactivity is unlikely to have been an important influence on the measurement of fidelity, although it cannot be entirely discounted.

The process evaluation hoped to produce a comprehensive assessment of the mechanisms of change. However, the Likert-type questions on gossip, social approval, norms and the emotional motivators we employed produced a poor distribution of responses in this population and so could not be utilised as planned in mediation analysis [73]. More work is needed to develop tools that can capture the brain-based mediating factors that lead to changes in target behaviours so that mechanisms of change can be better investigated and understood in low-income settings.

## Conclusions

The process evaluation framework that we developed allowed us to gather much insight on the successes and failures of the innovative *Komboni Housewives* programme in Zambia. As we had specified a clear theory of change for the intervention, we were well placed to design and execute a process evaluation that explored intervention delivery, receipt and change mechanisms. Whilst measurement of intervention implementation was relatively straightforward, it proved harder to measure psychological mediators of change in this context. Further work is thus required to develop measures to ascertain whether emotional drivers such as *disgust*, *affiliation* and *nurture* are as central to behaviour change as the Behaviour Centred Design approach posits. Whilst it is not surprising that an intervention with low reach and low fidelity across multiple behaviours should have a limited effect on behaviour, the results underscore the need to pay more attention to the practical issues of delivering a sufficient dose of an intervention in challenging and diverse rural and peri-urban contexts. This raises further questions about what dose is deemed sufficient and how costly it may be to deliver it. Standardising the use of evaluation frameworks such as this could improve the process evaluation of complex multiple behaviour change interventions and the utility of their findings.

## Additional file

Additional file 1: Table S1. Dose Delivered and Reach for Each Intervention Component and Cluster. (DOCX 19 kb)

## Abbreviations

NHCs: Neighbourhood Health Committee (Volunteers)
ORS: Oral rehydration salts
ORT: Oral rehydration therapy
ToC: Theory of change
