# Supplementary material for: Disentangling the effects of a multiple behaviour change intervention for diarrhoea control in Zambia: a theory-based process evaluation
Source: Global Health. 2017 Oct 17;13:78. doi: 10.1186/s12992-017-0302-0 (PMC5645837; doi:10.1186/s12992-017-0302-0)
Supplement: Additional file 1: Table S1. — Dose Delivered and Reach for Each Intervention Component and Cluster. (DOCX 19 kb) [file 12992_2017_302_MOESM1_ESM.docx]

**Additional file 1: Table S1. Dose Delivered and Reach for Each Intervention Component and Cluster**

|  |  |  | **ORT CORNER SESSIONS** | | | | | **WOMEN'S FORUMS** | | | |  | **ROAD SHOWS** | | | **RADIO** | | **OVERALL REACH** |
| --- | --- | --- | --- | --- | --- | --- | --- | --- | --- | --- | --- | --- | --- | --- | --- | --- | --- | --- |
|  |  |  | **Dose** | | **Reach** | | | **Dose** | | **Reach** | | **Dose** | **Reach** | | | **Dose** | **Reach** |  |
| **Cluster** | **Setting** | **Clinic Catchment Pop** | **Total No. Sessions Held** | **Mean No. Sessions per Day** | **Total No. Recipients** | **% Target Pop Attended Corner^1^** | **% Target Pop Attended Prize Draw^1^** | **Total No. Forums Held** | **Mean No. Forums per Day** | **Total No. Recipients (% from target pop)** | **% Target Pop Attended^1^** | **Order of Road Shows** | **Estimated Total No. Recipients** | **% Total Pop Attended^2^** | **% Target Pop Attended^1^** | **Total No. Call-in Shows^3^** | **% Target Pop Heard Radio Show^1^** | **% Target Pop Attended ≥1 Face-to-Face Event^1^** |
| 1 | Peri-urban | 46766 | 178 | 1.41 | 1339 | 1.8% | 0% | 23 | 1.9 | 342 (94.4) | 3.5% | 3^rd^ | 1900 | 9.9% | 5.0% | 7 | 48.5% | 14.0% |
| 2 | Peri-urban | 112695 | 193 | 1.55 | 1091 | 8.3% | 3.3% | 23 | 1.9 | 400 (93.3) | 23.3% | 1^st^ | 1500 | 28.1% | 1.8% | 8 | 36.2% | 35.0% |
| 3 | Peri-urban | 94290 | 195 | 1.52 | 1461 | 6.7% | 6.0% | 21 | 1.9 | 315 (90.5) | 11.7% | 4^th^ | 1500 | 8.5% | 0 | 4 | 18.2% | 23.3% |
| 4 | Peri-urban | 160077 | 231 | 1.82 | 2112 | 7.0% | 0% | 22 | 1.8 | 443 (80.8) | 17.5% | 2^nd^ | 2000 | 7.2% | 8.9% | 6 | 17.6% | 24.6% |
| 5 | Semi-rural | 26720 | 158 | 1.25 | 1222 | 5.3% | 5.6% | 21 | 1.9 | 374 (96.5) | 9.3% | 6^th^ | 2200 | 9.0% | 28.6% | 5 | 34.5% | 28.0% |
| 6 | Semi-rural | 31976 | 174 | 1.38 | 1024 | 18.6% | 14.8% | 22 | 1.7 | 373 (99.5) | 28.6% | 5^th^ | 1600 | 22.5% | 16.0% | 6 | 40.9% | 61.4% |
| 7 | Rural | 13715 | 137 | 1.08 | 678 | 27.4% | 26.4% | 12 | 1.0 | 230 (98.3) | 27.4% | 7^th^ | 1200 | 51.2% | 38.7% | 6 | 47.8% | 66.1% |
| 8 | Rural | 9955 | 120 | 0.95 | 517 | 23.1% | 13.5% | 14 | 1.0 | 246 (99.6) | 19.2% | 8^th^ | 1700 | 139.2%* | 42.3% | 8 | 35.3% | 61.5% |
| **Overall** | | **496,194 1386** | | **1.37** | **9444** | **12.3%** | **8.7%** | **158** | **1.64** | **2723 (96.1)** | **17.6%** |  | **13,600** | **34.5%** | **17.6%** |  | **34.9%** | **39.3%** |

*ORT Corner sessions (and Prize Draws) were delivered at clinics and recipients originated from throughout the clinic catchment area. Reach of ORT Corner sessions within the target population is therefore lower for ORT Corners than Women's Forums which were delivered within the communities inhabited by the target population. Attendance was not documented at the prize draws. ORT Corners ran Monday to Friday in each site, while forums ran from Monday to Friday and rotated between sites which is reflected in calculation of the number of events held per day.*

*^1^ Reach estimated using endline survey data from two random samples used to measure intervention outcomes: i) caregivers of children under-five with recent diarrhoea; ii) caregivers of infants under six months-of-age.*

*^2^ Reach estimated using data from a census conducted in the intervention areas during the intervention period. The target population of the road shows was the whole community so reach is reported for the total population.*

*^3^ Radio shows aired three times a week throughout the six-month intervention period. Radio call-in shows required the radio DJ to contact a women's forum and numbers do not therefore reflect total airtime.*

** Road show may have attracted individuals from other communities or there may have been an issue with the denominator.*
